# Host-Pathogen Interaction as a Driver of Cellular Senescence: Microbial Triggers and Host Response

**DOI:** 10.3390/ijms27146497

**Published:** 2026-07-22

**Authors:** Florin Iordache, Alexandru Andrei Zaharie, Petronela Mihaela Rosu, Alina Maria Holban, Carmen Curutiu

**Affiliations:** 1 Faculty of Veterinary Medicine, University of Agronomic Sciences and Veterinary Medicine of Bucharest, Splaiul Independentei 105, 050097 Bucharest, Romania; floriniordache84@yahoo.com (F.I.); alexandrei28022001@gmail.com (A.A.Z.); petronela.rosu@fmvb.usamv.ro (P.M.R.); 2 Department of Microbiology, Faculty of Biology, University of Bucharest, Splaiul Independentei 91–95, 050095 Bucharest, Romania; alina.m.holban@bio.unibuc.ro

**Keywords:** cellular senescence, oxidative stress, bacteria–host interactions, pathogen induced immunosenescence

## Abstract

Despite extensive research, the complex relationship between cellular senescence, aging, and host–pathogen interactions remains incompletely understood. This paper aims to review cellular and molecular alterations of senescent cells and critically examine the role of bacterial infections as key drivers in immunosenescence. Molecular mechanisms underlying pathogen-induced stress responses and the subsequent impact on host tissues and immune function are also highlighted. The novelty of this work lies in integrating current knowledge into direct and indirect mechanisms by which bacterial pathogens induce senescence, including genotoxic effects, oxidative stress, and disruption of host signaling pathways. Particular emphasis is placed on how bacterial virulence factors modulate critical pathways such as p53–p21, p16^INK4a^–Rb, NF-κB, mTOR, and cGAS–STING, thereby promoting a pro-inflammatory senescence-associated secretory phenotype (SASP) and facilitating chronic infection and tissue damage. By linking microbial activity with cellular aging processes, this work offers a novel perspective on the contribution of infections to premature aging and age-related diseases and highlights potential therapeutic targets for modulating senescence and improving host resilience.

## 1. Introduction

Cellular senescence indicates a state of stable cell cycle arrest in which proliferating cells become resistant to growth stimuli, usually in response to DNA damage. Leonard Hayflick originally defined senescence in 1965 after observing that human fetal fibroblasts finally stopped proliferating yet continued to be alive and metabolically active after extended time in culture. Senescent cells differ from terminally differentiated cells as well as quiescent cells, which could re-enter the cell cycle [1].

The questions of whether aging may be reversed and transformed into rejuvenation, as well as how aging differs from and is impacted by cellular senescence, remain unanswered. Furthermore, the significance of senescence in healthy and pathological aging as well as in life expectancy is still insufficiently understood [2,3].

If aging is associated with chronological age and with an overall physical and psychological journey through time being shaped by both your genetics and environmental factors, senescence is one of the hallmarks that focuses on the cellular, molecular, and genetic mechanisms that drive age-related diseases [3,4].

In diploid cells, senescence is an adaptive biological process that results from repeated cycles of cell division. There is a clear distinction between physiological senescence, involving telomere uncapping by repeated cell division (replicative senescence), and stress-induced premature senescence, also called “stasis”, triggered by a variety of external or internal stress factors such as oncogene activation (oncogene-induced senescence), chromatin and DNA disruptions (DNA damage-induced senescence), endo/exogenous oxidative stress, X-ray exposure or aggressive chemotherapy, endo/exogenous mitogenic signals, circulating angiotensin II [5,6,7,8], and microbial pathogens [9].

Senescent cells have been shown to have both beneficial and detrimental effects, depending mostly on the physiological setting, making the biological significance of senescence complex. For instance, senescence may be linked to a number of age-related disorders (osteoarthritis, atherosclerosis, pulmonary fibrosis, chronic kidney disease, diabetes, aged-related macular degeneration, Parkinson’s and Alzheimer’s diseases, and myelofibrosis), cancer, tissue degeneration, and inflammatory diseases, even though senescence has probably developed as a mechanism to prevent malignant transformation of damaged cells [10,11,12,13,14,15].

Bacteria play a dual and complex role in cellular senescence, acting both as drivers of premature aging in host tissues through chronic infection and, in some contexts, as beneficial agents that can delay it. Pathogenic bacteria induce senescence to create a favorable environment for their persistence, while some probiotic strains can help mitigate age-related deterioration. While some virulence factors produced by pathogenic bacteria can promote senescence by damaging the host DNA or increase ROS, some microbial metabolites such as short-chain fatty acids (SCFAs) produced by beneficial bacteria could exercise an anti-inflammatory response and promote antioxidant defenses [16].

Cellular senescence can be triggered by microbial pathogens via direct and indirect mechanisms. While direct mechanisms include genotoxins and pathogen-associated molecular patterns (PAMPs) and are responsible for genotoxic and oxidative stress, indirect mechanisms usually include signaling disruption and inflammatory signaling activation [17]. Bacterial pathogens that induce host-cell senescence play an important role in persistent infection, as cumulative cellular stress and a low-grade inflammatory environment can exhaust immune cells and impair the ability to clear the infection. Moreover, certain pathogens are capable of actively subverting host cellular machinery to induce a senescent phenotype, thereby promoting their persistence within host tissues, impairing regenerative processes, and contributing to the development of long-term pathological sequelae, including fibrosis and oncogenesis [18].

This review paper seeks to critically examine host–pathogen interactions as a key driver of cellular senescence, with particular emphasis on the molecular mechanisms underlying bacterial triggers and the corresponding host responses.

## 2. Modifications of Senescent Cells

Modification in senescent cells implies cellular and morphological modifications, molecular pathways, and gene expression alterations, chromatin/epigenetic changes, and appearance of a proinflammatory phenotype known as the *senescence-associated secretory phenotype (SASP)* [19].

Cellular and morphological features imply modifications of cell size and shape and changes in the architecture of the cell membrane and cytoskeleton, continuing with increasing the number of mitochondria, increasing the activity of lysosomal enzymes, and modifying the nuclear envelope [20].

### 2.1. Size and Shape Alterations

Senescent cells display an enlarged size and are flattened and irregularly shaped with enlarged and numerous nuclei due to the activation of the mTOR pathway. The plasma membrane plays a key role in maintaining the size and shape of the cells and also permits interactions with adjacent cells and extracellular space. Caveolin-1 protein is upregulated by p38 (MAPK pathway), thus inducing morphological alterations and influencing the adherence of senescent cells. Furthermore, a form of oxidized vimentin was also observed in the plasma membrane of senescent fibroblasts [21]. The mTOR (mechanistic target of rapamycin) is a serine/threonine kinase involved in signaling pathways stimulated by growth-promoting signals. There are two mTOR-containing multiprotein complexes (mTORC1 and mTORC2) with differential sensitivity to rapamycin and with different involvement in the regulation of cell growth. The mTORC1 complex (containing mTOR, mLST8, FKBP12, DEPTOR, and RAPTOR proteins) regulates signaling related to nutrients, growth, and stress factors, adjusting the balance between anabolism and catabolism, while the mTORC2 complex (mTOR, mSIN1, DEPTOR, and RICTOR proteins) orchestrates rearrangements of the cytoskeleton and activation of pro-survival pathways [22]. mTOR has been found in several subcellular compartments (plasma membrane, mitochondria, endoplasmic reticulum, lysosomes, and nucleus), with lysosomal mTOR regulating autophagy and lysosomal biogenesis [23]. mTORC1 stimulates plasma membrane depolarization by activating the Na^+^ channel Scn1a, which increases Ca^2+^ influx via NFAT/ATF3/p53 signaling, inducing senescence in preosteoblast cells [24]. Also, cytoskeletal actin and vimentin filaments, which have a major role in organelle anchorage, are regulated by mTOR- and CD42-dependent pathways, contributing to the altered shape of senescent cells [25,26].

### 2.2. Mitochondria Dysfunctions

Senescent cells frequently exhibit mitochondrial dysfunctions, due to altering ATP production which decreased inner membrane potential, reduced mitochondrial oxidative phosphorylation, followed by an excess of reactive oxygen species. A decrease in ATP synthesis lowers the AMP-ATP ratios, which activates 5′ AMP-activated protein kinase (AMPK), that triggers the mitochondrial autophagy catabolic pathway [27,28]. Additionally, AMPK limits glycolysis in senescent cells by promoting fatty acid oxidation, tricarboxylic acid cycle, and oxidative phosphorylation and causes cell cycle arrest by phosphorylating p53, which regulates the cells’ reaction to stress [29,30]. Dysfunctional mitochondria cannot be removed easily through mitophagy, their accumulation contributes to the installation of senescence-associated mitochondrial dysfunction (SAMD) [31].

### 2.3. Lysosomal Dysfunctions

An increased lysosomal volume is a sign of senescence because lysosomal proteins are overexpressed. Lysosomes are acidic organelles that contain a variety of hydrolytic enzymes that become more active in senescent cells like α-mannosidase, α-fucosidase, N-acetyl-β-hexosaminidase, and lysosomal β-galactosidase [32]. Increased production of LAMP1, the autophagy proteins ATG5 and ATG12, increased LC3 lipidation, and a decrease in the autophagy adaptor p62 are all stimulated by telomere shortening-induced senescence [33].

SASP is triggered by senescence-inducing stressors via mTORC1 signaling on hyperactivated lysosomes, which reinforces GMP–AMP (cGAMP) synthase (cGAS)/stimulator of interferon genes (STING)-mediated secretion [34]. A number of SASP lysosomal proteins, including gelsolin, metalloproteinase MMP2, and cathepsins B, D, and Z, are upregulated in age-dependent serum [35]. Senescence inducers such as DNA damage, oncogene activation, and replicative exhaustion can cause aberrant mTORC1 signaling through lysosomal accumulation of cholesterol mediated by cholesterol importer ABCA1 [36]. Ca^2+^ signaling activates TFEB/TFE3 (transcription factor EB/E3) in lysosomal biogenesis and transcriptional autophagy upregulation. Lysosomal Ca^2+^ release by TRPML1 channels activates TFEB through V-ATPase-dependent lysosomal ATG8 lipidation. Subsequently, the folliculin complex that is an activator of RagC/D required for mTORC1-mediated TFEB phosphorylation and suppression is sequestered [37,38].

### 2.4. Endoplasmic Reticulum Dysfunctions

Endoplasmic reticulum dysfunction promotes SASP remodeling in senescent cells. The build-up of unfolded or misfolded protein due to cellular stress in the endoplasmic reticulum is one reason for the increased translation rate. Moreover, the misfolded protein may aggregate, resulting in a breakdown of proteostasis or cellular homeostasis. The unfolded protein response (UPR) pathway is a highly conserved pathway that contains three key branches: IRE1 (inositol-requiring enzyme 1), ATF6 (activating transcription factor 6), and PERK (protein kinase R-like ER kinase) [39,40]. IRE1α catalyzes the cytosolic splicing of a 26-nucleotide intron from the mRNA of XBP1 (X-box binding protein 1), an effective transcription factor that drives the expression of numerous genes involved in proteostasis. Unfolded proteins translocate ATF6 to the Golgi apparatus, where it is cleaved by S1P and S2P proteases, generating and enhancing the expression of chaperones and ER-associated degradation components. Upon ER stress, PERK phosphorylates eIF2α, leading to transient inhibition of global protein synthesis [39]. In a recent study by Hetz and Dillin 2024, they showed that senescent cells reveal increased expression of ER stress-associated proteins, such as the chaperone BiP and ATF6 proteins, suggesting that senescent cells exhibit basal levels of ER stress [40]. IRE1α induces early senescence through RIDD (IRE1-dependent decay of mRNA) of specific pro-oncogenic factors in Ras-induced models, suggesting that UPR signaling promotes senescence in response to oncogenic stress, serving as a barrier to tumorigenesis [41]. Using DNA-damaging agents such as etoposide and irradiation to induce senescence, Payea et al. 2024 [42], found that senescent cells exhibit a strong and persistent phosphorylation of eIF2α. Despite this phosphorylation, translation of the downstream effector ATF4 is impaired due to decoupling of eIF2α phosphorylation from ATF4 translation, leading to failures in ribosome availability and function [42].

Furthermore, Puebla-Huerta et al. showed that doxorubicin/etoposide-induced senescence increased mitochondria-ER contact sites (MERCS) but reduced significant ER-mitochondria Ca^2+^ flux. Mechanistically, senescent cells exhibit decreased expression of IP3R and reduced interaction between type 1 IP3R and VDAC1, impairing Ca^2+^ transfer. In vitro and in vivo senolytic effects are demonstrated by inhibition of ER-mitochondria Ca2+ flux, providing a unique approach to target senescent cells [43].

### 2.5. Nuclear Dysfunctions

In senescent cells, the nuclear transport is altered. Nucleoporin Nup93 is lost in old and permeable nuclei, leading to a decreased signal of FG-Nups. Nup93 was shown to bind FG-Nups (peripheral nucleoporins with phenylalanine-glycine-rich repeats) located at the central channel, supporting the nuclear pore complexes’ diffusion barrier [44]. Research employing aged human fibroblasts revealed a decline in a number of nuclear transport factors, including RanBP1, CAS, and karyopherin a2, along with a corresponding decrease in the nuclear import rate of proteins [45].

Hutchinson–Gilford progeria, a premature aging disease, is associated with structural alterations in the nuclear lamina due to a mutant form of lamin A called progerin. Progerin expression reduces levels of histone H3 lysine 9 trimethylation (H3K9me3), disrupts the Ran gradient, inhibits the nucleus localization of Ubc9, and stops Tpr import, decreasing the nuclear import rate. The nuclear import protein transportin-1 (TNPO1) is sequestered by microtubules and mislocated in the cytoplasm, which affects the nuclear localization of its cargo proteins, Nup153 and hnRNPA1, in progeria patient-derived cells. Nup153 and Tpr are the Nups that form the basket located at the nuclear pore complex side [46,47].

Lamin B1 is a biomarker of senescence both in vitro and in vivo. Lamin B1 loss is not dependent on p38 MAPK, NF-κB, ATM kinase, and ROS signaling pathways that positively regulate senescent phenotypes. However, lamin B1 loss could be induced by activating either the p53 or pRB pathways, leading to its degradation more by a decrease in mRNA stability than by caspase-mediated degradation, as seen in apoptosis [48]. Laminin B1 forms a complex with the LC3 autophagy protein and mediates degradation upon Ras-induced oncogenic stress targeting of lamin B1 to the lysosome [47,49].

In many senescent cells, condensed chromatin, called senescence-associated heterochromatin foci (SAHF), with a well-organized structure, accumulates HP1, H3K9me3, and H3K27me3. SAHF regulates gene expression and cell cycle arrest upon oncogene-induced senescence [50].

Telomeres are the ends of chromosomes and are comprised of nucleoproteic complexes (hexanucleotide repeat TTAGGG associated with shelterin proteins such as TRF1, TRF2, RAP1, POT1, TPP1, and TIN2). Telomere stabilization is dependent on the interaction with lamin A/C and LAP2α [47,51]. In senescent cells, both LAP2α and lamin A/C are decreased. In Hutchinson–Gilford progeria, progerin reduces the levels of LAP2α and prevents the interaction of TRF2 with lamin A/C, leading to telomere attrition through TRF2 deregulation [52].

## 3. Molecular Pathways and Gene Expression Alterations

Cyclin-dependent kinases 4 and 6 (CDK4/6) play a critical role for cellular proliferation. These kinases are activated by D-type cyclins (D1, D2, and D3) and phosphorylate the retinoblastoma (RB) family of proteins. Cyclin-dependent kinase inhibitors such as p16^INK4a^, encoded by CDKN2A, and p21CIP can inhibit CDK4/6. Both p16^INK4a^ and RB1 are tumor suppressors, and their disruption results in tumor progression. The cell cycle pathways P16^Ink4a^/Rb and P19Arf/P53/P21Cip1 are key players in senescence [53,54].

Generally independent of the P19Arf/P53/P21Cip1 pathway, the P16^Ink4a^/Rb pathway is believed to be the primary pathway leading to senescence in cells. Cellular senescence caused by stress and oncogenic factors is associated with the P16^Ink4a^/Rb pathway, but in human cells, these two pathways are co-regulated. In response to stress agents, the P16^Ink4a^ protein expression increases, possibly by reducing the inhibitory protein of Ink4, such as Bmi-1 expression. The P16 protein (encoded by CDKN2A) is localized in the nucleus, and its amino-terminal end binds to CDK4/6, inhibiting the phosphorylation of the major substrate retinoblastoma protein Rb. Unphosphorylated Rb sequesters E2F transcription factors, preventing E2F from moving the cells from the G1 phase to the S phase and ultimately leading to cell senescence [55,56].

The p53 protein is a tumor suppressor protein, inactivated in most tumors and up-regulated in senescent cells. The ubiquitin ligase MDM2 (murine double minute 2), which is overexpressed in some common tumors, can promote the degradation of p53 by related proteases or directly inhibit the activity of p53 protein. The P19^Arf^ protein encoded by the Arf gene locus overlaps with the Ink4a gene locus and can bind to and inhibit MDM2 activity, thereby participating in the p53 pathway. When DNA damage occurs (ionizing radiation or telomere dysfunction), P19Arf is upregulated, inhibiting MDM2 activity and activating p53. Following p53 activation, P21^Cip1^ (encoded by CDKN1A) inhibits both CDK4/6 and CDK2, which inhibits RB phosphorylation and thus blocks the cells in the G1 phase and causes cellular senescence [57,58].

### 3.1. Chromatin and Epigenetic Changes

*Senescence-associated heterochromatin foci (SAHF)* are dense heterochromatin foci that contain the high-mobility group A proteins (HMGA1 and HMGA2), histone variants mH2A, H3K9me3, H4K20me3, H3K27me3, and HP1 protein but are not a universal feature of all types of senescent cells [59,60].

Late-passage cells reaching senescence display reduced expression of the core H3 and H4 histones, and the linker histone H1 is depleted in senescent cells [61]. During cellular senescence, downregulation of EZH2 causes a loss of H3K27me3 and the activation of p16. The Polycomb repressive protein, a long noncoding RNA called ANRIL, has an important role in mediating the repression of p16 [62]. The loss of H3K4me3 in senescent cells is mediated through the cleavage of the histone H3 tail by cathepsin L proteases and through the demethylation of H3K4 by JARID1A and JARID1B demethylases [63]. In oncogene-induced senescence, the loss of H3K9me3 and the gain of H3K4me3 are found to activate BMP2 via the BMP-SMAD pathway [64].

Chromosome centromeres are heterochromatic regions, displaying structural alterations in senescence. These regions contain repeats named satellites, which are normally constitutively repressed, but in replicative senescent cells, pericentric satellite HSATII distends and becomes accessible. This modification is named *senescence-associated distension of satellites (SADS)* and was confirmed via fluorescent in situ hybridization (FISH) experiments on pericentric satellite II and centromeric alpha satellite [65]. Mobile genetic elements, retrotransposons, normally repressed in heterochromatin, have also been revealed to increase chromatin accessibility in senescent cells [66].

**Figure 1:**
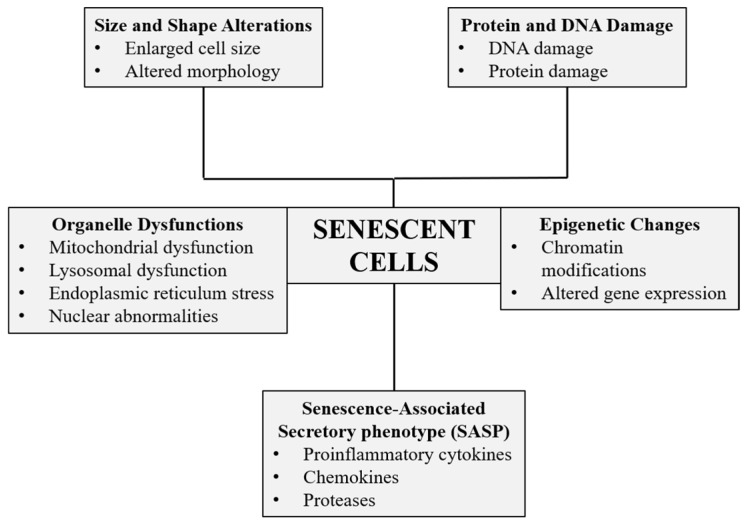
Cellular and molecular modifications of senescent cells.

Acetylation of the H3K14 histone in mesenchymal stem cells is regulated by SIRT1 deacetylation when its elevation is closely associated with stem cell senescence [67]. Furthermore, acetylation at H4K16 histone disrupts telomeric silencing and triggers cellular senescence [68]. The p300/CBP establish acetylation marks at loci such as H3K27 and H4 histone, facilitating the activation of senescence drivers like p16. In endothelial progenitor cells, the p300/Sp1 complex enhances H4 acetylation and accelerates senescence [69]. Deacetylases (HDACs), in particular, the sirtuin family (SIRTs), play a major role in maintaining chromatin homeostasis and reducing cellular senescence. SIRT1 removes acetyl groups from H3K14 to suppress transcription of senescence-related genes [70]. HDAC inhibitors increase acetylation and activate the p21 gene, thereby inducing senescence. However, HDAC3 and HDAC4 can delay senescence by binding transcription factors like Yin Yang 1 (YY1) or Zinc-binding Protein 89 (ZBP-89) and suppress p16 expression [71,72]. The most important modifications of senescent cells are shown in Figure 1 below.

### 3.2. Senescence-Associated Secretory Phenotype (SASP)

DNA damage responses (DDRs) are a key initiator of the pro-inflammatory state of the SASP due to the activation of various signaling pathways such as NF-κB, p38 MAPK, mTOR, and cGAS–STING, but there are also certain SASP molecules that are secreted independent of the DDR. Depending on intrinsic and extrinsic factors and the moment, the SASP composition is highly dynamic and includes interleukins, chemokines, growth factors, proteases, lipids, exosomes, and small non-coding DNA [73].

### 3.3. Interleukins

IL-1α, IL-1β, IL-6, and IL-8, are the most studied SASP factors. IL-1α is cleaved by caspase 5 or caspase 11, moving from the cell surface to the nucleus in a manner dependent on copper, calcium, or calcium-binding protein S100A13 and activating transcription for other pro-inflammatory factors. Furthermore, in senescent cells, miR-146a and/or miR-146b function downstream of IL-1α to control IL-6 and IL-8. IL-11 is activated using SIRT1 deacetylase and is associated with senescence, vitamin D deficiency, and pulmonary fibrosis. IL-33 is secreted by the senescent hepatic stellate cells and can promote hepatocellular carcinoma development [74,75].

### 3.4. Chemokine

CCL2 is regulated by IL-1α and IL-1β and has a strong chemoattractant effect for monocytes and macrophages. CCL2 is negatively regulated by BMI1 protein through an IL-1α-mediated mechanism in senescent mesenchymal stem cells. CCL5 is associated with non-small-cell lung cancer progression and senescence in theca and interstitial cells. CXCR2 mediates senescence via p38-MAPK and p53 downstream; CXCL1–CXCR2 via NF-κB subunit RelA (p65); CXCL10-CXCR3 signaling in natural killer cells; and CXCL11 secreted by endothelial cells is associated with breast cancer aggressiveness [76,77].

### 3.5. Growth Factors

Transforming growth factor-β (TGFβ) is secreted by senescent cells in a model of aged mice with a fractured callus, delaying healing of tibial bone fractures. By paracrine secretion, TGFβ can promote SASP upregulation and activate immune cells. Growth differentiation factor 15 (GDF15), another member of the TGFβ superfamily, promotes osteoarthritis from senescent chondrocytes. Hepatocyte growth factor (HGF) is a SASP factor secreted by senescent fibroblasts [78,79].

### 3.6. Bioactive Lipids

SASP can express leukotrienes with pro-inflammatory or pro-fibrotic effects if cysteinyl leukotriene receptors exist on the surface of interacting cells. Prostaglandins, following their metabolic conversion to the oxylipin dihomo-15d-PGJ2, can contribute to reinforcing senescence-associated proliferation arrest [80,81].

### 3.7. Exosomes

Small extracellular vesicles were identified in various types of senescent cells. Exosomes (30–150 nm) are heterogeneous vesicles that can incorporate proteins, lipids, microRNAs, and other factors. Recent studies showed that exosomes from senescent cells promote cancer cell proliferation [73,82]. Table 1 below shows the most common senescence biomarkers.

## 4. Immunosenescence

The concept of immunosenescence was proposed by Roy Walford in the 1960s, who explained the diminished ability of immune cells to respond to pathogens, with a reduced vaccine efficacy and an increased risk of age-related diseases. Immunosenescence is regulated by several signaling pathways, such as mTOR, NF-κB, JAK-STAT, melatonin, sirtuin, cGAS-STING, and AMPK pathways, whose alterations lead to aberrant immune reactions and increased sensitivity to age-related diseases. Exposure to ultraviolet radiation, reactive oxygen species (ROS), mitochondrial dysfunction, increased glycolysis, smoking, alcohol, pollution, lack of exercise, and other factors contribute to the accumulation of damage in various components of the immune system, including the innate immune system and the acquired immune system [83,84].

*Upregulated signaling pathways in immunosenescence are NF-κB, mTOR, JAK-STAT, and cGAS-STING*

*Transcription factor NF-κB* can be activated during cellular damage and stress. Genetic depletion or chemical inhibition of NF-κB decreases DNA damage and stress in aged mice, delaying cellular senescence and age-related symptoms. Persistent NF-κB activation drives chronic inflammation (inflammaging), which impairs immune surveillance, reduces T cell diversity, and promotes tissue degeneration. NF-κB suppresses autophagy via mTOR, a key inhibitor of autophagic flux. Impaired autophagy in senescent cells leads to the accumulation of injured mitochondria and protein aggregates, which activate oxidative stress and the inflammasome. Furthermore, NF-κB upregulates anti-apoptotic proteins, preventing the clearance of senescent cells and contributing to cancer risk during aging [85,86,87].

*The mTORC1 pathway* is associated with cellular senescence. In aged mice treated with metformin or everolimus that inhibits mTORC1, the level of IL-2 increased, proliferation of T cells increased, and oxidative stress in CD4^+^ T cells decreased. Moreover, in elderly humans, inhibition of mTORC1 with RAD001 and BEZ235 reduced infection rates over 12 months, indicating an enhancement of immune function [87,88].

mTORC2 prevents lipid peroxidation and mitochondrial ROS accumulation via activation of AKT and inhibition of GSK3β, thereby accompanying their long-term survival. The inhibition or genetic deletion of SGK1, the downstream effector of mTORC2, promotes the formation of memory precursors of CD8^+^ T cells and enhances their long-term survival [87,89].

During aging, the *JAK-STAT signaling pathway* can be dysregulated, contributing to immunosenescence. Hyperactivation of STAT3 stimulates secretion of proinflammatory cytokines, such as IL-6 and IL-23, promoting SASP and sustaining inflammation. JAK3 mutations determine defective T cells and natural killer (NK) cell maturation, weakening immune responses against infections and tumors. Overactivation of STAT3 promotes T helper 17 (Th17) cell expansion and suppresses Treg function. Moreover, JAK-STAT activation disrupts hematopoietic stem cell differentiation, advancing myeloid rather than lymphoid lineage commitment [87,90].

*The cGAS-STING pathway senses* cytosolic DNA damage, mitochondrial dysfunction, or nuclear envelope disruption. Through the cGAMP–STING–TBK/IKK axis, it induces NF-κB-dependent expression of inflammatory cytokines such as IL-6 and CXCL10, promoting SASP and a decreased expression of IFN-I, resulting in decreased presentation of antigens to T lymphocytes [34,87,91].

*Downregulated signaling pathways in immunosenescence are the AMPK, Melatonin, and Sirtuin pathways*

*AMPK* is a pivotal serine/threonine protein kinase that regulates cellular energy metabolism. AMPK is a central energy sensor that extends lifespan by promoting autophagy via mTOR inhibition and ULK1 activation. This pathway leads to stimulation of mitochondrial function through SIRT1 signaling, improving the NAD+/NADH balance, suppressing inflammatory responses, and modulating stress resistance via the FOXO3/p53 pathways. Moreover, AMPKα1 is essential for CD8^+^ T cell memory, and, in CD4^+^ T cells (the CD27^−^CD28^−^ subset), AMPK activation under stress or DNA damage induces p38 autophosphorylation, leading to telomerase suppression and proliferative arrest [87,92].

*Melatonin* plays a pivotal role in regulating circadian rhythms, significantly decreasing in level with age. Melatonin suppresses proinflammatory cytokines (e.g., IL-1β, IL-6, TNF-α, and FN-γ) and enhances anti-inflammatory cytokines (IL-4 and IL-10) by inhibiting the NF-κB pathway, although the effects are dose- and pathology-dependent. Melatonin scavenges ROS and upregulates the expression of antioxidant enzymes superoxide dismutase (SOD) and glutathione peroxidase (GPX), reducing oxidative damage and SASP immune cells. Furthermore, in immune cells, melatonin enhances CD4+/CD8+ T cell proliferation and antigen responsiveness and modulates the Treg/Th1/Th2 balance, endorses macrophage polarization toward the M2 phenotype, and amplifies NK cell cytotoxicity via SIRT1 pathway activation. As a circadian regulator, melatonin stabilizes clock genes, reduces cortisol-mediated immunosuppression, and coordinates rhythmic immune cells [93,94,95].

The *Sirtuin pathway* contains a family of proteins that play crucial roles in immune aging by regulating mitochondrial metabolism, oxidative stress, and NF-κB signaling in various immune cell types. In hematopoietic stem cells, sirtuin 3 (SIRT3) preserves genomic stability, increases superoxide dismutase 2 (SOD2), and maintains mitochondrial integrity, delaying cellular senescence. SIRT1 and SIRT6 mitigate inflammatory responses in macrophages by inhibiting NF-κB signaling and suppressing excessive TNF-α and IL-1β expression. In dendritic cells, SIRT1 modulates autophagy and cytokine secretion, enhancing antiviral responses. In NK cells, SIRT2 and SIRT6 suppress NK cytotoxicity by downregulating glycolysis and mitochondrial respiration. Furthermore, both SIRT1 and SIRT7 regulate B cell class-switch recombination (CSR) and influence immunoglobulin maturation. Overall, the sirtuin family acts as a critical regulator of immune aging, providing a new therapeutic target [70,87,96].

Innate immune senescence is characterized by a reduction in the ability to process and present antigens, which results in a diminished reaction to stimuli. Adaptive immune senescence is linked to a loss of TCR diversity and impaired immunological memory formation. Impaired immune cells adopt a SASP that affects tumor and other age-related disease progression, supporting the importance of these subsets of cells [83,97].

The aberrant signaling pathways during aging determine dysfunction in almost all kinds of immune cells, ranging from hematopoietic stem cells (HSCs) to mature immune cells.

In HSCs from older mice, inhibition of IL1 increased the myeloid lineage, and degeneration of sympathetic nervous system signaling through adrenoreceptor β3 resulted in premature HSC aging [83,87].

*Natural killer (NK) cells* are involved in the recognition of infected and malignant target cells and are characterized by HLA class I-specific receptors of the KIR and NKG2 gene families. Although this repertoire remained remarkably stable until old age, in a minority of subjects, a breakdown of NK cell repertoire diversity was observed that might influence immune surveillance and healthy aging. Aged NK cells present a reduced degranulation capacity and an impaired perforin secretion, possibly linked to alterations in Ca^2+^-dependent exocytosis via Munc13-4, a key protein in NK cell granule release. Aging leads to the downregulation of key transcription factors such as EOMES and T-bet, impeding NK cell maturation [87,98].

*Macrophages* can be clustered into pro-inflammatory, antitumorigenic M1, and anti-inflammatory, pro-tumorigenic M2 subsets. The existence of senescent macrophages in vivo remains debatable. With aging, the ability of macrophages to phagocytose pathogens decreases, antigen-presenting capacity decreases, expression of Toll-like receptors is downregulated, and the expression of Treg cells is increased. In senescent macrophages, expression of p16^INK4^ reduced LPS-induced IL-6 expression by inhibiting the AP-1 pathway [83,99]. During aging, the monocyte subset CD14+CD16+ is elevated with downregulated expression of CX3CR1, whereas the CD14+CD16− subset is decreased. The level of β2-microglobulin in plasma increases, leading to a proinflammatory phenotype in monocytes, increasing the production of IL-6 and TNF-α via Toll-like receptor 2/6 (TLR2/6) signaling but inhibiting the production of IL-10 and transforming growth factor-β (TGF-β1) [76,87].

*T cells* contain various subpopulations with heterogeneous changes in senescence. With time, naïve T cells decrease due to thymic involution, diminishing the diversity of the TCR repertoire and reducing the ability to respond to novel antigens. Senescent T cells, especially CD8^+^ T cells, contribute to the SASP by secreting pro-inflammatory cytokines such as IL-6 and TNF-α and promoting an inflammatory environment. In senescent CD8^+^ T cells, the expression of surface markers changes clearly, with a drastic reduction of CD28 and a high expression of CD57, Tim-3, killer cell lectin-like receptor subfamily G member 1 (KLRG-1), and CD45RA. Senescent T cells present an upregulation of inhibitory receptors PD-1 and CTLA-4, limiting the cytotoxic activity and capacity to clear infections or malignant cells [82,87]. The senescent CD27^−^ CD28^−^ CD8+ T cell population displays features such as a reduced proliferation rate, shorter telomeres, and increased levels of γH2AX and p38. The retrotransposon LINE-1 is upregulated in senescent cells, leading to a type 1 IFN response modulating T cell quiescence, effector function, and exhaustion [99,100].

Aging also affects T helper (CD4^+^) cell function, specifically by affecting the balance between Th1 and Th2. The dominant subpopulation became primarily Th2 involved, in humoral immunity. Th1 cells become functionally impaired with consequences in activating macrophages and clearing intracellular pathogens. Furthermore, the regulatory T cell (Treg) compartment undergoes age-related changes, showing impaired control over immune activation, contributing to chronic inflammation and autoimmunity during aging [100,101].

*B cells* are cells that develop an immunological memory after infection or vaccination by transforming into plasma cells that produce protective antibodies. In immunosenescent B cells, the E47 mRNA (E47 transcription factor involved in B-cell maturation) degradation rate is significantly increased, as shown in the in vivo study on older mice; this also correlates with defects in antibody secretion and immunoglobulin class switching [102,103]. B cell activation and differentiation are controlled by TLR signaling, especially for IgM^+^ memory B cells. In vitro studies have shown that TLR7 and TLR9 promote IgM secretion [104]. Furthermore, aging B cell activation is impaired via downregulation of CD40, leading to a reduced responsiveness to B cell receptor stimulation [105].

*Dendritic cells (DCs)* are special cells that can derive from both lymphoid stem cells and myeloid stem cells and play a vital role as professional antigen-presenting cells. In aged DCs, the capacity to phagocytose antigens and migrate is significantly impaired, and MHC and CD40 expressions are downregulated, weakening their ability to release proinflammatory cytokines (Table 2). Furthermore, in aging HSC, the WNT/CDC42 pathway is activated, leading to impaired differentiation of plasmacytoid dendritic cells (Table 2). Bone marrow-derived DCs display increased IL-23 levels and increased p19 mRNA expression via TLR activation [87,106,107].

## 5. Mechanisms of Bacteria-Induced Host Cellular Senescence

Between chronic infections and accelerated aging, there is a strong connection. The effects may be the results of the direct activity of the pathogens themselves or the prolonged immune response of the host to their action [18]. Bacterial contributions to host cellular senescence are mainly mediated by virulence factors and stress-inducing molecules that trigger direct DNA damage and oxidative stress and by indirect mechanisms also targeting host inflammatory signaling.

### 5.1. Direct Mechanisms

They are usually represented by host DNA damage by bacterial genotoxins and cellular ROS induction.

*Bacterial genotoxins* are bacterial metabolites or virulence factors that manipulate DNA of mammalian host cells. The effects of genotoxins include nuclear enlargement, DNA repair response, cell cycle arrest in the G2/M phase, and/or cell death. When the DNA repair system is overwhelmed, the cell can enter senescence or apoptosis.

Cytolethal distension toxins (CDTs) are produced by many Gram-negative bacteria, typhoid toxin produced by *Salmonella typhi*, and colibactin produced mainly by strains of *Escherichia coli* are the main characterized genotoxins [108].

Both CDTs and typhoid toxin are virulence factors consisting of two components: the enzymatic A subunit and the binding B subunit.

CDTs are type AB2 toxins, consisting of one A subunit (CdtB) and two B subunits (CdtA and CdtC). The active subunit (CdtB) acts like DNase, entering the host nucleus to create DNA double-strand breaks. This triggers a DNA damage response that arrests the cell cycle and causes cell distension. CDTs’ intoxication produced progressive cell distension and cytotoxicity in cultured eukaryotic cells. The host reply to toxins is different depending on the target cells: in T-cell and B-cell lines, apoptosis of the cells was reported, while in other types of cell cycle arrest in the G1 and/or G2 phases was observed [109].

These toxins are produced by numerous pathogenic bacteria involved in oral, gastrointestinal, or systemic diseases. For example, some Gram-negative bacteria such as *Escherichia coli*, *Campylobacter jejuni*, *Helicobacter pylory*, or *Shigella dysenteriae* produce and secrete cytolethal distending toxins (CDTs) with potentially genotoxic effects [110].

An important role of these genotoxins is to disrupt the host immune response. In a study conducted by Mathiasen et al. (2021) [111] on the toxin CDT (cytolethal distending toxin), it was observed that it affects CD4 T lymphocytes, essential for adaptive immunity. The toxin causes senescence of activated CD4 T cells in laboratory conditions and possibly in the body. Senescent T cells acquire a special behavior called SASP (senescence-associated secretory phenotype). This phenomenon is partly controlled by the ATM–p38 signaling axis. The results suggest a link between bacterial infections and T cell senescence, highlighting the role of genotoxins in modulating the immune system [111].

The typhoid toxin produced by *Salmonella typhi* has an A2B5 structure and contains two A subunits (CdtB and PltA) and five B subunits (PltB). PltA has an ADP ribosyltransferase activity, and CdtB has a DNase activity. Similar to the A subunit of CDTs, typhoid toxin subunit CdtB targets the host cell DNA and causes a DNA repair response, cell cycle arrest, or cell death. contributing to the development of typhoid fever. Upon internalization, the toxin is secreted in the lumen of the *Salmonella*-containing vacuole and then packaged in small vacuoles that exit the infected cells to release the typhoid toxin in the extracellular medium and intoxicate the cells in autocrine and paracrine manners [109].

Colibactin is a metabolite produced by the phylogenetic group B2 strains of *E. coli* and other bacteria. It causes severe DNA damage, including double-strand breaks and chromosomal abnormalities. Unlike CDT, the action of colibactin requires direct contact between the bacterium and the host cell, such as adhesion or invasion in host cells, because it is an unstable molecule. Colibactin produced by *Escherichia coli* targets host DNA directly. Genotoxin enters intestinal cells and causes DNA double-strand breaks and cross-links between DNA strands. This causes stress replication and genomic instability. This triggers the ATM-p53-p21 signaling pathway, resulting in irreversible cell cycle arrest. Affected cells may enter a state of senescence, secreting growth factors that can stimulate the proliferation of other surrounding cells. This is associated with infections and even the development of colorectal cancer.

In conclusion, bacterial genotoxins are important virulence factors that affect the DNA of host cells, having a major role in pathogenicity and the development of serious diseases [108].

Another central mechanism underlying senescence is *oxidative stress*, predominantly driven by elevated levels of reactive oxygen species (ROS), which induce mitochondrial DNA damage, regulate redox-sensitive signaling pathways, and initiate the senescence-associated secretory phenotype. Reactive oxygen and nitrogen (RON) species are continuously produced in the body under both normal and pathological conditions. The main source of intracellular ROS is mitochondrial respiration, where electron leakage during oxidative phosphorylation generates superoxide and other reactive molecules. In addition, multiple enzyme systems (e.g., NADPH oxidases, xanthine oxidase, nitric oxide synthases) and cellular compartments contribute to ROS production. External factors such as pollutants, radiation, drugs, and dietary components can further increase ROS/RNS levels. Moreover, immune cells generate high amounts of ROS/RNS during defense responses, contributing to both host protection and oxidative stress [112].

*Helicobacter pylori*, a major cause of chronic gastritis and peptic ulcers, induces persistent cellular senescence mainly in gastrointestinal epithelial cells, even after the infection is eradicated. This effect is driven primarily by inflammation-induced ROS, which cause DNA double-strand breaks, telomere shortening, and genomic instability. These processes activate stress pathways (e.g., PARP-1) and are further enhanced by virulence factors such as CagA, which promote oxidative damage. Additionally, *H. pylori* triggers a senescence-associated secretory phenotype (SASP), characterized by increased inflammatory cytokines (e.g., IL-6, IL-8) through NF-κB activation. Beyond the gastrointestinal tract, this senescence may also affect skin cells, contributing to chronic inflammatory skin diseases [18].

Another bacterial species, *Pseudomonas aeruginosa*, induces cellular senescence through the secretion of some virulence factors, such as pyocyanin and ExoU, which promote mitochondrial dysfunction and the accumulation of ROS. These elevated ROS levels function as signaling mediators that drive immune cells toward premature senescence, thereby leading to a sustained impairment of their capacity to fight infections. In patients with burn wounds or chronic ulcers, the presence of pyocyanin creates a collection of senescent cells that fail to replicate or migrate, impeding tissue regeneration and encouraging excessive scarring [113].

Even *E. coli* colibactin acts as a genotoxin and induces DNA double-strand breaks and increased ROS. ROS production contributes to the maintenance of lesions and the entry of cells into senescence.

Pneumolysin (PLY), a pore-forming toxin produced by *Streptococcus pneumoniae*, can trigger cellular senescence in glial cells (such as microglia) by inducing membrane pores that disrupt mitochondrial function. This initiates a cascade where excessive ROS are produced that subsequently activates MAPK (ERK1/2, JNK, and p38) and NF-kB signaling pathways. These pathways modulate cell-cycle regulatory proteins (such as p53, p21, and p16), forcing the cell into an irreversible growth arrest. Consequently, senescent glia produce elevated levels of pro-inflammatory factors that sustain chronic inflammation and neurodegenerative pathologies [114].

It is worth mentioning that both DNA damage and the production of ROS are factors involved not only in senescence but also in aging.

DNA damage can be induced in several ways, including activation of oncogenes (RAS and BRAF), inhibition of tumor suppressors (CSNK1A1, PTTG, and PTEN), DNA damage (e.g., oxidative stress, alkylating drugs, radiotherapy, and environmental radiation), and dysfunctional and shortened telomeres. With every cycle of cell division, DNA damage occurs at the telomere ends. Accumulation of DNA breaks will activate tumor suppressor protein p53. CDKN2a encodes the two isoforms, p14^ARF^ and p16^ink4a^; p14^ARF^ inhibits Mdm2, which prevents the degradation of p53, leading to cell cycle arrest. p16^ink4a^ inhibits CDK4/6, which causes hypo-phosphorylation of Rb, allowing the Rb-E2F complex to stay intact, inhibiting S phase, and leading to senescence. In senescence and apoptosis, the p53 pathway is active, acting as a cellular gatekeeper regulating cell growth and division or protecting the body from cancer by pausing the cell cycle to allow for DNA repair or by triggering apoptosis. In cancer, the p53 pathway is inhibited, leading to the accumulation of mutations and, ultimately, to cancer [115].

Production of ROS due to high cellular metabolism, SASP, chemotherapy, and radiotherapy activates specific pathways such as p38 MAPK pathways leading to senescence or p53, p27, and p21 pathways leading to apoptosis. In cancer the ROS activates other pathways such as PIK3/Akt for proliferation, HIf-1α, for angiogenesis, and ERK1/2 for cytoskeleton remodeling. In cancer, the presence of ROS is also stimulated by tumor microenvironment factors such as chronic inflammatory molecules, high glycolysis, hypoxia, and pro-oncogenic factors [116].

### 5.2. Indirect Mechanisms

The indirect mechanisms of bacterial-induced host cellular senescence rely on signaling disruption, the development of cytokine storms, and chronic NF-kB activation [18].

Bacteria can promote cellular senescence through disruption of the host signaling pathways, driving cells into an irreversible state of growth arrest while simultaneously inducing a pro-inflammatory phenotype. This microbial subversion of host cellular processes contributes to accelerated tissue aging and the persistence of chronic inflammatory responses [17].

Persistent exposure to bacterial components drives cells into senescence indirectly by pushing host tissues into a chronically exhausted, hyper-inflammatory state.

The process unfolds in three major stages:

(a) PAMP Activation: Continuous stimulation by bacterial pathogen-associated molecular patterns (PAMPs), such as lipopolysaccharides (LPS), activates host pattern-recognition receptors, most notably TLR4 [17].
(b) NF-κB Axis Dysregulation: Sustained TLR4 signaling amplifies the NF-κB and p38 MAPK pathways, elevating expression of the cyclin-dependent kinase (CDK) inhibitors p16^INK4A^ and boosting transcription of pro-inflammatory cytokines, including IL-6 and IL-8 [114].
(c) Paracrine Propagation: Secreted IL-6 engages CXCR2 receptors on neighboring cells, creating a positive feedback loop that spreads the senescence-inducing inflammatory signal throughout surrounding tissue [117].

Bacterial interference reshapes host cell metabolism, redirecting resources toward pathogen persistence and trapping the cell in a dysfunctional metabolic state [18]. This shift is reinforced through ROS production. Intracellular bacteria usually elevate mitochondrial reactive oxygen species (ROS), creating oxidative stress [118]. Excess ROS overstimulates the Akt/mTOR signaling axis, promoting cellular hypertrophy and pushing the host cell toward premature senescence. Then, intense intracellular infection and mitochondrial damage can release nuclear or mitochondrial DNA into the cytosol, activating the cGAS–STING pathway. This further amplifies NF-κB–driven inflammation and enhances pathogen immune evasion [119,120].

Following the disruption of key signaling pathways, senescent cells initiate secretion of the senescence-associated secretory phenotype (SASP). This secretome is characterized by markedly elevated concentrations of pro-inflammatory interleukins (e.g., IL-1, IL-6), chemokines, and tissue-degrading matrix metalloproteinases (MMPs). Under chronic pathological conditions, SASP activity contributes to progressive tissue fibrosis, functional exhaustion of immune cell populations, and structural deterioration of affected tissues [18,121].

Bacterial effectors responsible for premature cellular senescence by targeting and disrupting core host signaling networks differ among pathogens. For example, bacterial genotoxins [111,122], effector proteins [123,124], type III secretion systems (T3SS) [124,125], soluble virulence determinants, quorum-sensing (QS) molecules [126,127,128,129], pyocyanin [127], and exotoxins [130] interfere with host cell signaling, may induce immunosenescence, and manipulate host cell death pathways.

The molecular effectors responsible for premature host cell senescence of most investigated bacterial pathogens, together with the manipulated host signaling pathways, are summarized in Table 3 below.

### 5.3. Inflammasome Signaling

Inflammasomes are protein complexes serving as critical molecular triggers that induce inflammatory responses. Inflammasomes recognize damage-associated molecular patterns (DAMPs) and pathogen-associated molecular patterns (PAMPs), leading to the secretion of pro-inflammatory cytokines such as IL-1β and IL-18, as well as the initiation of pyroptosis, a form of cell death [148].

Gut microbiome, fungus, viruses, free cholesterol, and ceramide changes create dysbiosis and altered microbial metabolites influencing inflammation by the NLRP3 inflammasome. Also, ROS, monosodium urate, high uric acid and glucose, α-synuclein, extracellular adenosine triphosphate, and DNA fragments activate NLRP3, and nucleotide-derived metabolites promote inflammation by the NLRC4 inflammasome due to aging signals [149]. High glucose induces macrophage senescence and SASP factors via phosphorylation of NLRC4, which further activates the NF-κB/Caspase-1 cascade through the IRF8-dependent pathway [150].

The architecture of the inflammasome is built using *sensor molecules* (NLRP1, NLRP2, NLRP3, NLRP6, NLRC4, and NLRP12, AIM2), *adaptor molecules* (ASC), and *effector molecules* (caspase-1).

NLRP3 is considered a central driver of inflammaging and is expressed in immune and parenchymal cells, and its activation requires a two-step process: a priming step (via NF-κB signaling from Toll-like receptors) that upregulates NLRP3 and a trigger step (mitochondrial dysfunction, lysosomal rupture, or ionic flux) that promotes the NLRP3 sensor to assemble with the ASC adaptor and caspase-1, leading to the release of inflammatory cytokines such as IL-1β and IL-18. Chronic NLRP3 activation is believed to sustain the inflammation of aging [151]. The canonical inflammasome pathway is caspase-1 dependent, but in recent years, caspase-4/5/11-dependent noncanonical NLRP3 activation has also been reported. Caspase-4/5 in humans and caspase-11 in mice bind with lipopolysaccharide in both monocytes and nonmonocytes, leading to autoproteolysis of the proteins. The alternative NLRP3 activation pathway depends on the TLR4–TRIF–RIPK1–FADD–CASP8 signaling pathway, and, in human monocytes, apolipoprotein C3 activates this caspase-8-dependent alternative NLRP3 inflammasome pathway through TLR2 and TLR4 [152].

AIM2 is the only one to be able to activate caspase-1 in response to the infection of specific pathogens such as HSV and *Francisella tularensis*, *Streptococcus pneumoniae*, and *Legionella pneumophila*, leading to panoptosis (concomitant activation of pyroptosis, apoptosis, and necroptosis) as a protection mechanism for the host. AIM2 inflammasome activation is a type I interferon pathway [153].

NAIP/NLRC4 contains the NLR family of apoptosis inhibitory proteins (NAIPs). NLRC4 proteins are observed in murine models; human cells can only encode one type of NAIP protein. NAIPs contain a Baculovirus inhibitor-of-apoptosis repeat (BIR) and bacterial proteins such as flagellin. PrgJ induces NAIP2-mediated NLRC4 oligomerization and activation. Once NAIP proteins are activated, they can interact with other inactivated NLRC4 and induce activation of caspase-1.

Gram-negative bacteria such as *Salmonella typhimurium* can activate the NAIP/NLRC4 inflammasome by T3SS proteins, suggesting its importance for immune responses against Gram-negative pathogens. In *Helicobacter pylori* infections, NLRC4-deficient mice control the bacterial burden better than wild-type animals, suggesting that the function of the NAIP/NLRC4 inflammasome is pathogen specific [154]. Furthermore, bacterial and host damage-associated molecules can trigger an inflammasome response not only in immune cells but also in other cell types, such as epithelial cells, that start to secrete proinflammatory cytokines Il-β and IL-18 [155].

## 6. Strategies Against Premature Senescence

Preventing premature senescence requires a multifactorial approach, combining lifestyle optimization, antioxidant and microbiome-based strategies, and emerging senotherapeutics. While lifestyle and nutrition remain the most accessible and evidence-supported interventions, future therapies targeting senescent cells directly hold strong potential for extending health periods. Different lifestyle interventions, including exercise, nutrition, intermittent fasting, consumption of phytochemicals, prebiotics and probiotics, and adequate sleep, can produce anti-senescence effects in model organisms and humans [156].

**Figure 2:**
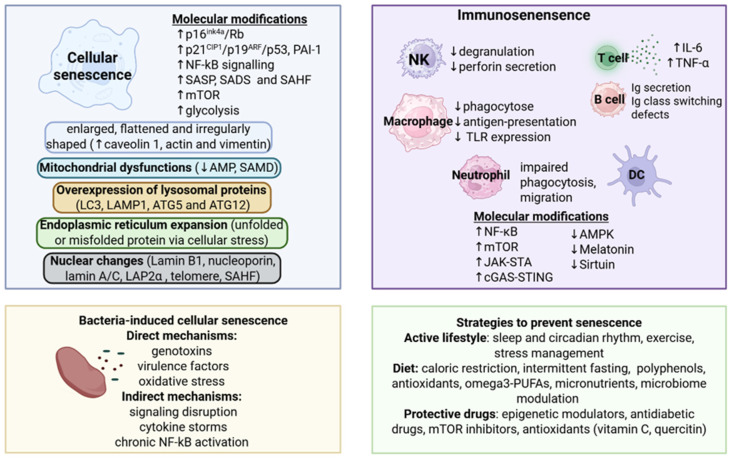
Illustration of the most important cellular and molecular traits of senescence, particularities of immunosenescence and bacteria-induced host cell senescence, along with efficient strategies proposed to prevent early senescence (up arrow—upregulated, down arrow—downregulated) Created in BioRender. Ditu, L. (2026) https://BioRender.com/9ynjup2.

Diet plays a major role in modulating cellular senescence and the aging process by influencing metabolic pathways, oxidative stress, and inflammation. Nutritional factors can accelerate or delay the onset of senescence, depending on the quality and long-term effects on cellular homeostasis. It has been observed that caloric restriction, without malnutrition, has been consistently associated with delayed cellular aging, reduced oxidative stress, and decreased accumulation of senescent cells. This effect is mediated through several key signaling pathways that regulate metabolism (mTOR, AMPK, and sirtuin pathways), autophagy, and DNA repair. In terms of composition, diets high in saturated fat, refined sugars, and processed foods have been observed to promote oxidative stress and chronic inflammation, while Mediterranean-type diets rich in antioxidants, polyphenols, vitamins, and omega-3 fatty acids help attenuate oxidative stress and reduce oxidative damage. Among polyphenols, resveratrol and quercetin modulate signaling pathways involved in cell survival and inflammation. Vitamins C and E are antioxidants that protect against DNA and protein damage. Diet also impacts immune aging (immunosenescence) by modeling the inflammatory state and immune cell function. Poor nutrition exacerbates chronic inflammation (“inflammaging”), while balanced diets can preserve immune competence and reduce the burden of senescent immune cells [157,158]. Regular physical activity practices improve mitochondrial function but also improve sleep and reduce stress that indirectly limits chronic inflammation and DNA damage [159]. The diversity and composition of microbiota are also important, as aging is accompanied by significant changes in the gut microbiome, notably a decline in microbial diversity and an increase in pro-inflammatory species, both of which are associated with functional deterioration and chronic disease. Recent research indicates that microbial metabolites—such as short-chain fatty acids, urolithins, and bile acids—contribute to the regulation of inflammation, preservation of epithelial barrier integrity, and modulation of metabolic processes (Figure 2). Importantly, interventions aimed at restoring microbial homeostasis—including dietary modification, probiotic/prebiotic supplementation, personalized nutrition, and fecal microbiota transplantation—have shown encouraging results in mitigating microbial aging, supporting tissue integrity, and delaying senescence in both experimental models and human studies [160].

As biomedical strategies, the development of senolytics and senomorphics offers complementary approaches either by clearing harmful senescent cells or by modulating their pro-inflammatory and degenerative secretory profiles. Senolitics, such as tyrosine kinase inhibitors or flavonoids, selectively eliminate senescent cells, while senomorphics suppress SASP without killing cells, reducing inflammation, and tissue dysfunction. These compounds are promising therapeutic agents, improving tissue function, and extending health span in diverse animal models [161].

## 7. Conclusions

Cellular senescence is a key interface between aging, immunity, and infection, with bacterial pathogens playing a critical and often underappreciated role in driving premature cellular aging and disease progression. The complex interplay between host aging and microbial factors highlights the potential of microbial balance as essential elements in the development of preventive measures against cellular senescence and age-related morbidity. Accumulating evidence suggests that age-related shifts in bacterial communities—both in composition and function—can contribute to chronic inflammation, immune dysregulation, and metabolic imbalance, all hallmarks of senescence. At the same time, certain beneficial bacteria may help delay these processes by supporting immune homeostasis and tissue health. Thus, bacteria are not merely passive inhabitants but active participants in aging, capable of both accelerating and mitigating senescence depending on their balance and interactions with the host. Understanding how microorganisms influence cellular senescence provides essential insights into the progression of age-related diseases and potential therapeutic targets.

## Figures and Tables

**Table 1:** The main biomarkers of senescence.

Protein/Marker	Role in Senescence
SA-β-galactosidase	Marker of lysosomal dysfunction
Lipofuscin	Marker of lysosomal dysfunction
p53	Activation triggers cell cycle arrest
Rb (retinoblastoma protein)	Inhibition triggers cell cycle arrest
p21^CIP1^	Inhibits cyclin-dependent kinases
p16^INK4A^	Inhibits phosphorylation of pRb
Bcl-2	Increased expression inhibits apoptosis
macroH2A1.1	Marker of SAHF
macroH2A1.2	Marker of SAHF
H3K9Me2/3)	Marker of SAHF
HP1 (heterochromatin protein 1)	Marker of SAHF
Pericentric satellite HSATII	Marker of SADS
Phospho-histone H2A.X (Ser139)	Marker of DNA damage
Lamin B1	Reduced expression, disruption of nuclear envelope
HMGB1 (high mobility group box 1)	SASP component
IL-6 (Interleukin 6)	SASP component
TNF-α (tumor necrosis factor α)	SASP component
MMP3 (matrix metalloproteinase-3)	SASP component

**Table 2:** The main mechanisms of the immune cell control of senescence.

Immune Cells	Role in Senescence
HSC self-renewal	Decrease, functional decline with reduced self-renewal
Myeloid lineage	Increase, promote myeloid differentiation from HSC
Lymphoid lineage	Decrease, inhibit lymphoid differentiation from HSC
Thymic progenitor	Decrease, accumulation of late-differentiated, senescent-like T cells
Naive T cell	Decrease due to thymic involution
T cell diversity	Reduce, secreting pro-inflammatory cytokines such as IL-6 and TNF-α
B cell diversity	Reduce, defects in antibody secretion and immunoglobulin class switching.
NK cells	Decrease by reduced degranulation capacity and impaired perforin secretion,
Neutrophil function	Decrease, impaired function
M1 macrophages	Increased secretion of inflammatory molecules
M2 macrophages	Decrease, pathogen phagocytosis decreases, antigen-presenting capacity decreases, expression of Toll-like receptor is downregulated
Dendritic cells	Decrease, impaired capacity to phagocytose antigens and migrate

**Table 3:** Bacterial effectors involved in host-signaling disruption related to senescence induction.

Bacterial Pathogen	Effector(s) Responsible for Cellular Senescence	Disrupted Host Signaling Network(s)	Ref.
*Helicobacter pylori*	Cytolethal Distending Toxin (CDT), CagA	DNA damage response (DDR); p53–p21 and p16INK4a–Rb pathways	[122]
*Salmonella* spp.	SopE, SopE2, SopB; mitochondrial-targeting effectors	cGAS–STING; Rho GTPases; PAK; Abl; STAT3; apoptosis pathways	[123,124]
*Pseudomonas aeruginosa*	Pyocyanin; 3-oxo-C12-HSL; Exotoxin A; ExoS, ExoT, ExoU	ROS signaling; PGC-1α; AhR; Rho GTPases; caspase-4 pathways; cytoskeleton regulation	[126]
*Escherichia coli*	Colibactin (genotoxin)	DNA double-strand break response; ATM/ATR; p53 pathway	[131]
*Shigella flexneri*	IpaB, IpaJ, VirA	NF-κB modulation; MAPK pathways; cytoskeleton dynamics; apoptosis evasion	[132]
*Chlamydia trachomatis*	CPAF (chlamydial protease-like factor); inclusion proteins	p53 degradation; host cell cycle control; apoptosis inhibition pathways	[133,134]
*Neisseria gonorrhoeae*	Opa proteins; LOS (lipooligosaccharide)	EGFR signaling; MAPK; oxidative stress pathways; immune response	[135,136]
*Mycobacterium tuberculosis*	ESAT-6; CFP-10; ROS-inducing factors	NF-κB; MAPK; mitochondrial stress; autophagy and apoptosis pathways	[137,138]
*Staphylococcus aureus*	Leukotoxins (LukA); protein A vesicles	NF-κB–p53–p21 axis; ROS-mediated signaling; inflammatory pathways	[139,140]
*Streptococcus pneumoniae*	Streptolysins; hydrogen peroxide	DDR; p53–p21; p16^INK4a^; ROS-mediated Golgi and immune signaling disruption	[114,141]
*Listeria monocytogenes*	Listeriolysin O (LLO); ActA	Mitochondrial stress; ROS; NF-κB; autophagy	[142,143]
*Bacillus anthracis*	Lethal toxin (LF); edema factor (EF)	MAPK signaling; cAMP pathways; immune signaling suppression	[144,145]
*Clostridium difficile*	TcdA; TcdB (toxins A and B)	Rho GTPases; cytoskeleton signaling; inflammation and apoptosis pathways	[146,147]

## Data Availability

No new data were created or analyzed in this study. Data sharing is not applicable to this article.
